# You’re carrying so many people’s stories: vicarious trauma among fly-in fly-out mental health service providers in Canada

**DOI:** 10.1080/17482631.2022.2040089

**Published:** 2022-02-23

**Authors:** Candace Roberts, Francine Darroch, Audrey Giles, Rianne van Bruggen

**Affiliations:** a Department of Health Sciences, Carleton University, Ottawa, ON, Canada; b School of Human Kinetics, University of Ottawa, Ottawa, ON, Canada; c Northern Counselling and Therapeutic Services, Ottawa, ON, Canada

**Keywords:** Vicarious trauma, fly-in/fly-out, mental health, mental health service provider, rural and remote, Inuit

## Abstract

**Purpose:**

The purpose of this article is to examine the factors that influence fly-in and fly-out (FIFO) mental health service providers’ experiences of vicarious trauma as they deliver services to communities in Inuit Nunangat through a constructivist self-development theory (CSDT) lens.

**Method:**

Using a participatory action research methodology, we conducted eight semi-structured interviews with providers to understand their perspectives on the risk of developing vicarious trauma and potential mitigation strategies.

**Results:**

We identified three themes through thematic analysis: 1) vicarious trauma is a risk associated with working in communities with high rates of trauma; 2) establishing individual and organizational strategies to reduce risk of vicarious trauma may improve FIFO providers’ well-being and career longevity; and 3) FIFO models of care may offer protective benefits for mental health service providers against vicarious trauma.

**Conclusions:**

We conclude that FIFO models of care may help mental health service providers to manage the risk of vicarious trauma through reduced caseload and less time spent in community.

## Introduction

Mental health service delivery to rural and remote regions of Canada is complex as community members face barriers to mental health care (Mental Health Commission of Canada (MHCC), n.d.). Individuals residing in communities in Inuit Nunangat, which comprises four regions that include the Inuvialuit Settlement Region, Nunavut, Nunavik, and Nunatsiavut, are often forced to travel to obtain mental health services or rely on transient and fly-in fly-out (FIFO) healthcare providers and who do not reside in their community (Inuit Tapirit Kanatami, 2014; Inuit Tapiritt Kanatami, 2021; Oosterveer & Young, 2015). FIFO healthcare providers who live in southern Canada rotate in and out of Inuit regions (Inuit Tapiritt Kanatami, 2021), which can engender its own self of problems as these providers may not be familiar with Inuit culture or language (Inuit Tapirit Kanatami, 2014). Recruiting and retaining permanent health professionals is challenging due to the remoteness of Inuit communities (Inuit Tapirit Kanatami, 2014; Oosterveer & Young, 2015). This is especially problematic for Inuit who reside in Inuit Nunangat and may experience poor health outcomes related to mental health (Inuit Tapirit Kanatami, 2014), which are symptoms of colonialism, racism, and marginalization amongst other challenges that result in persistent inequality (Inuit Tapirit Kanatami, 2014; Kirmayer et al., 2011). For mental health service providers to provide continuity in services to Inuit, there must be considerations for the providers’ mental health and well-being. Constant turnover of mental health providers is a challenge for clients who need continuity of care (Inuit Tapirit Kanatami, 2014). Although there is limited literature that examines the experience of vicarious trauma in FIFO service providers, there is an overwhelming amount of evidence that experiences of trauma can be shared between provider and client (Finklestein et al., 2015; McCann & Pearlman, 1990; Pearlman & Mac Ian, 1995; Quitangon, 2019). While researchers have examined the effects of vicarious trauma on therapists globally (Cohen & Collens, 2013; Finklestein et al., 2015; Harrison & Westwood, 2009), to our knowledge, there is limited peer-reviewed research on the impact for FIFO mental health service providers Inuit communities in Canada. As such, our objective is to examine the factors that influence the experience of vicarious trauma for FIFO mental health service providers delivering services to Inuit Nunangat using the CSDT framework.

## Literature review

### Mental health service delivery in rural and remote Canada

Members of communities in rural and northern regions of Canada face significant difficulties when accessing mental health care (Dyck & Hardy, 2013). Although rural and remote communities are often categorized by geographic isolation, they can also be defined by their organizational, social, and cultural arrangements (Bourke et al., 2013). Most often, the terms rural and remote are used under the same umbrella (Bourke et al., 2013), though Pitblado (2005) found that the term “northern” is also frequently used in the context of rural health research in Canada. Barriers that affect access to services for these residents include limited local mental health care services and clinicians, high travelling costs, and long wait times, among others (Boydell et al., 2006; Dyck & Hardy, 2013). Due to these challenges, many communities in the Circumpolar North rely on short-term locum providers who travel from outside the region to provide healthcare services (Huot et al., 2019). Wakerman et al. (2012) described FIFO services in a healthcare setting in a variety of ways: specialist outreach services; “hub-and-spoke” or outreach arrangements for allied health and specialists, which consists of an establishment (hub) providing services to multiple secondary establishments (spokes); “orbiting staff” who spend 12 months or more in one or two specific communities; long-term shared positions in which practitioners service the same communities (e.g., one month on/one month off); and short-term locums who service numerous rural locations on a short-term basis. In Canada, little is known about FIFO mental health services to Inuit communities, although mental health service delivery is particularly important for Inuit, who have complex histories of colonization, trauma, marginalization, and racism (Kirmayer et al., 2011). For FIFO mental health professionals working with Inuit, managing and monitoring symptoms of vicarious trauma is essential given the magnitude of historical and intergenerational traumatic issues experienced by this population and thus treated by these health care providers.

## Experiences of trauma for inuit

Inuit living in Inuit Nunangat experience many indicators of poor health (Inuit Tapirit Kanatami, 2014). Efforts of assimilation such as forced relocations, residential school and federal day school trauma, killing of sled dogs, and other challenges (Kirmayer et al., 2011) have resulted in historical trauma and a loss of cultural cohesion (Brascoupé & Waters, 2009). As a result, Inuit experience disproportionate burden of disease and inequalities in healthcare services for this population (Kirmayer et al., 2011; Nelson & Wilson, 2017). Maintaining awareness of the historical and ongoing impacts of colonization and marginalization is fundamental to understanding the trauma that Inuit have experienced and continue to experience.

## Vicarious traumatization

Secondary exposures to trauma can negatively affect the well-being of mental health providers and the quality of care they are able to provide (Quitangon, 2019). Three frequently used constructs that have been used interchangeably to describe the impact of providing trauma therapy on mental health providers are: compassion fatigue, secondary traumatic stress syndrome, and vicarious trauma (Devilly et al., 2009; Quitangon, 2019; Voss Horrell et al., 2011). These three constructs are interrelated and complex, with some arguing that they measure the same phenomenon (Devilly et al., 2009), while others argue that they in fact relate to different phenomena (Jenkins & Baird, 2002; Quitangon, 2019). Quitangon (2019) described compassion fatigue, which was originally referred to as secondary trauma stress syndrome, as empathic strain and exhaustion from caring for people in distress, which present as PTSD-like symptoms. Burnout is mentioned frequently in the literature as a persistent state of exhaustion that is a result of prolonged exposure to occupational stress (Quitangon, 2019). Although both constructs describe manifestations of exhaustion, unlike vicarious trauma, neither compassion fatigue nor burnout are specific to working with clients who have experienced trauma (Quitangon, 2019). There is a body of research that supports vicarious trauma as the only concept that describes a change in world views that are similar to the changes that occur in the traumatized client and that emphasizes cognitive symptomatology (Jenkins & Baird, 2002; Pearlman & Mac Ian, 1995; Quitangon, 2019).

Pearlman and Mac Ian (1995) defined vicarious trauma as the inner transformation of a trauma provider due to a cumulative and empathetic engagement with the client’s traumatic experiences (Pearlman & Mac Ian, 1995). Clemans (2005) described vicarious trauma in a broader manner and conceptualized it as emotional, physical, and spiritual transformations that may occur when working with members of populations that have experienced trauma. Documented symptoms of vicarious trauma include changes in one’s identity, worldview, interpersonal relationships, sense of oneself in the world, cognitive schemas or a person’s beliefs and assumptions about the world (Pearlman & Saakvitne, 1995), and post-traumatic stress disorder (PTSD)-like symptoms such as intrusive imagery, painful experiences of images, and emotions that are parallel to the traumatic memories of clients (Jenkins & Baird, 2002). For the purposes of this paper, aligning with the definitions provided by Jenkins and Baird (2002), Pearlman and Mac Ian (1995), and Quitangon (2019), we will consider vicarious trauma as a separate construct that occurs as a result of interactions with clients who have experienced trauma. Considering the complexity of trauma caused by historical and ongoing impacts of colonialism experienced by Inuit, it is important to explore the experiences of vicarious trauma among FIFO mental health providers who empathetically engage with Inuit.

## Risk factors for mental health service providers

Mental health providers are particularly vulnerable to vicarious trauma as a result of repeated exposure to details of clients’ trauma experiences (Quitangon, 2019). Researchers have found that the more time mental health professionals spend with their traumatized clients and the heavier the workload, the higher the providers’ risk of developing vicarious trauma (Finklestein et al., 2015; Pearlman & Mac Ian, 1995). Additional influences on vicarious trauma include stressful client behaviours, the nature of the clientele, work setting, and social-cultural context, as well as provider characteristics such as personal history of trauma, professional development, and current stressors and supports (Pearlman & Mac Ian, 1995). In a study that examined the predictors of vicarious trauma in non-victims, Lerias and Byrne (2003) reported that young age, being female, low socioeconomic status, high levels of stress, and low levels of social support may predict vulnerability to vicarious trauma. These risk factors may be relevant for FIFO mental health providers serving Inuit because of the unique circumstances in which they deliver services.

## Mitigation of vicarious trauma

To mitigate the risks of vicarious trauma for mental health service providers, it is essential to understand the protective and supportive factors. Cohen and Collens (2013) conducted a metasynthesis to examine the impact of trauma work on trauma workers, and they posited that personal factors such as optimism and spirituality can be considered resilience factors due to their impact on practitioners’ ability to cope with work-related distress. Optimism was found to not only function as a coping strategy, but also as an aspect of posttraumatic growth. Other researchers have suggested that personal strategies such as the balancing of personal and work life can moderate the negative impact of trauma work (Pearlman & Mac Ian, 1995). These findings are in contrast to findings from a study conducted by Bober and Regehr (2006), who found that although clinicians who work with victims of violence reported the usefulness of engaging with coping strategies such as self-care and leisure activities, there was no association found between self-care and lower traumatic stress scores.

Bober and Regehr (2006) proposed that the most significant predictor of high trauma scores for mental health service providers was the number of hours worked per week with clients who experience clients; organizations should thus consider the ways in which they distribute workload to providers to limit their traumatic exposure (Bober & Regehr, 2006). Cohen and Collens (2013) found that organizations can play instrumental roles in managing the distress of mental health workers, and these authors recommended that organizations foster a systematic approach to managing the impact of trauma work by providing institutional support and encouraging individual coping strategies.

Finally, numerous professional variables have been identified as affecting the development of vicarious trauma such as mental health provider training and professional support (Finklestein et al., 2015; Michalopoulos & Aparicio, 2012; Trippany et al., 2004). In a study looking at the development of vicarious trauma among social workers, Michalopoulos and Aparicio (2012) determined that more professional experience and increased support predicted a decrease in vicarious trauma symptoms. Having access to peer support systems, specialized trauma education, and training to support professional self-efficacy can strengthen provider resources and help to manage their risk of developing vicarious trauma (Finklestein et al., 2015; Trippany et al., 2004). These personal, professional, and organizational factors are important for FIFO mental health workers serving Inuit Nunangat and the organizations that employ them to consider as these variables can help reduce the likelihood of developing vicarious trauma.

## Traumatization of mental health staff in rural and remote Canada

Research and available information on the practice and experience of delivering mental health services in northern Canada is limited (O’Neill et al., 2013). Furthermore, the literature surrounding the transmission of trauma and coping strategies for mental health providers in the North is scant (O’Neill, 2010). In a review looking at secondary trauma in mental health practitioners in northern communities, O’Neill (2010) demonstrated the connection between isolated mental health practice and secondary trauma. In their review, secondary trauma was defined under the constructs of vicarious trauma, burnout, compassion fatigue, and secondary trauma stress. The author highlighted the vulnerability of providers to the various constructs of secondary trauma by describing the potential impact of professional and personal isolation combined with the requirements of empathic engagement with clients (O’Neill, 2010). In a study looking at the experiences of rural and remote nurses in Canada, Jahner et al. (2020) detailed the risks of encountering distressing incidents that affect the psychological health and physical safety. Participants in their study reported concern over limited protective strategies and a lack of supportive action from organizations, which put providers’ psychosocial health and safety (Jahner at al., 2020). It appears that traumatization occurring for mental health providers in rural and remote is two-fold: stress from working remotely with limited support, and a response to the trauma experienced by individuals living in remote areas.

Factors specific to northern mental health practice may contribute to the development of secondary trauma. O’Neill (2010) listed several contributing factors: prolonged interaction with traumatic material, lack of clinical supervision, high visibility in small communities, and complex overlapping relationships. Although, as noted above, researchers have identified protective practices to prevent vicarious traumatization of mental health providers such as access to supervision (Finklestein et al., 2015; Pearlman & Mac Ian, 1995), organizational support (Cohen & Collens, 2013), and training (Finklestein et al., 2015; Harrison & Westwood, 2009; Pearlman & Mac Ian, 1995), O’Neill (2010) acknowledged that these practices are not always possible in northern communinities. Although outside of a FIFO context, O’Neill recognized the difficulties in developing supportive social networks in isolated or rural communities in combination with a lack of clinical supervision, leaving providers vulnerable to personal and professional isolation. Indeed, this is problematic given the extensive number of scholars who have noted how support can reduce the risk of vicarious trauma. The purpose of the current study was to extend the literature to include experiences of vicarious trauma among FIFO mental health providers who deliver services to Inuit communities in Inuit Nunangat. Through our participatory action research (PAR) with Northern Counselling and Therapeutic Services (NCTS), we aimed to co-identify and recommend strategies to reduce the risk of traumatization for FIFO mental health service providers.

## Theoretical framework

Vicarious trauma has its theoretical basis in the constructivist self-development theory (CSDT), which allows researchers to conceptualize an individual’s adaptation to trauma through interaction between a traumatic event, personal history, and the social and cultural context (Pearlman & Mac Ian, 1995). The underpinning of this theory is that individuals construct their realities through cognitive schemas or perceptions (Trippany et al., 2004). Trippany et al. (2004) noted that the CSDT framework proposes that changes in perceptions or cognitive schemas can occur as a result of interacting with clients’ traumatic material and personal characteristics. The new information and experiences presented by clients are incorporated into the beliefs and systems of meaning for trauma therapists, which encourages change within the provider. There are five components of the self that are affected by exposure to trauma: frame of reference; self-capacities; ego resources; psychological needs; and cognitive schemas, memory, and perception. According to CSDT scholars, vicarious trauma reactions and distorted beliefs occur within the five components (Trippany et al., 2004). By using a CSDT lens, we sought to better understand how FIFO mental health service providers experience vicarious trauma. To our knowledge, this theoretical framework has not been explicitly discussed in the context of FIFO practitioners who serve Inuit Nunangat.

## Methodology

The research was approved by Carleton University’s Research Ethics Board (CUREB-B 112643). This study was conducted using a PAR approach in partnership with NCTS. NCTS offers a variety of services across the North including in-person counselling services, crisis response, clinical staff fill in, among others. In accordance with a PAR approach, the aim of this research was to co-create action and change with participants, specifically through changes to existing policy, procedures, and practice guidelines within the partner organization to optimize their service provision in the North (Baum et al., 2006). An advisory board, which consisted of two FIFO mental health service providers, two researchers, and one representative in a management role from our partner organization, guided all phases of the research, which was refined and changed to reflect advisory board members’ insights. The two FIFO mental health service providers were employed by NCTS, and took part in the research study. While one researcher did not have any experience related to FIFO service provision, the other researcher lived remotely in northern Canada for 17 years and was offered health care services through a FIFO model. Both researchers provided guidance on the research process, while the other members offered their guidance on the interview guide and the preliminary results, which they helped to finalize. One of the members of the advisory board served as a co-author on manuscripts stemming from the research. Members of the advisory board met virtually eight times by Zoom. All advisory board members were located in southern Canada as a result of the COVID-19 restrictions in place at the time the study was conducted (i.e., research in the territorial north was halted by the territorial governments). Communication was maintained over the course of the research study through email and virtual meetings so that advisory board members could provide ongoing feedback and advice regardless of location.

Using an interview guide co-created with the advisory board, the first author conducted eight semi-structured interviews with FIFO mental health service providers who work with NCTS and deliver services to communities in Inuit Nunangat to understand the experiences of vicarious trauma among FIFO mental health providers (see, Table I: Service Providers). Given the small population of FIFO providers servicing Inuit communities in Canada, pseudonyms were assigned and no detailed information about participants was provided to preserve the anonymity of participants. We recruited participants who met the following eligibility criteria: spoke English and had a minimum of one year of experience with FIFO delivery of mental health services to residents of communities in Inuit Nunangat. We recruited participants through an email that was circulated within the partner organization and snowball sampling (Ghaljaie et al., 2017). Snowball sampling is particularly helpful when recruiting members of populations with specific characteristics (Ghaljaie et al., 2017). After conducting interviews with the first two participants, who were members of the advisory board, snowball sampling was used to connect with other mental health service providers. Before conducting interviews, each participant provided written informed consent. In total, there were six female participants and two male participants, all of whom identify as Caucasian and reside outside of Inuit Nunangat. While participants all reported a minimum of ten years of counselling experience, their work in the FIFO capacity ranged from one to ten years. Time spent in Inuit communities was described as dependent on the type of contract, which differed among providers, varying from a few weeks for crisis response and short-term staff fill in, to multiple months for school settings and longer-term staff fill in. Participants received a $25 gift card to thank them for their involvement in the research.

**Table I.** Service providers

Pseudonym	Years of FIFO Service Provision to Inuit Nunangat
Susan	10 years
James	10 years
Diane	1 year
Tanya	2 years
Carol	7 years
Rebecca	5 years
David	2 years
Rachel	3 years

[insert Table I]

Each interview was conducted remotely using Zoom software or telephone. Two interviews were conducted using Zoom, while six interviews were conducted using the telephone. We digitally recorded all interviews, which ranged in length between 45 and 90 minutes. The semi-structured interview guide included questions such as, *Do you believe that there is compassion fatigue or vicarious trauma among mental health providers? What do you or your colleagues do to mitigate the risk of vicarious trauma? What supports are available for practitioners who experience vicarious trauma? What motivates you to return to northern communities to provide mental health services?* We transcribed the interview data verbatim and the first author checked the transcription accuracy before sending the transcripts to participants for verification. Participants made few changes: two participants changed their transcripts were to remove identifying information, and one participant added additional information for clarification. We then uploaded the transcripts into a qualitative software data analysis programme, NVivo^10^, for coding and analysis.

## Analysis

To analyse the transcripts, the first and second author used Braun and Clarke’s (2006) six-step approach to thematic analysis using an inductive approach. We also engaged in reflexivity while following Braun and Clarke’s (2019) update to data analysis. The first and second author familiarized themselves with the data by reading the transcripts. Then, the authors proceeded to generate initial codes and assign descriptive data segments. From the data segments, the authors began to organize the data to develop potential themes. The authors reviewed the potential themes for consistency and differences, and to determine if they were relevant to the extracted data. To ensure the themes captured the experiences of FIFO service providers, the findings were shared with the advisory board members who helped the authors refine the themes. Reviewing the results and discussing the findings was an iterative process shared with the advisory board. In alignment with a PAR approach, theme construction required cycles of participation, action, and reflection. Thus in the final step, which Braun and Clarke’s (2006) referred to as “developing the essence” (p. 22), we constructed three themes with the advisory board: 1) vicarious trauma is a risk associated with working in communities with high rates of trauma; 2) establishing individual and organizational strategies to reduce risk of vicarious trauma may improve FIFO providers’ well-being and career longevity; and 3) FIFO models of care may offer protective benefits for mental health service providers against vicarious trauma.

To engage in reflexivity, we reflected on our positionalities as they related to the research, such as how our own experiences of trauma and our professional work may impact how we perceive the experience of vicarious trauma. For example, all authors have undergraduate or graduate level training in psychology, and thus may have preconceived ideas of risk and protective factors for the development of vicarious traumatization. In addition to following Braun and Clarke’s (2006) approach, engaging with the CSDT framework informed the interpretation and analysis of the data by allowing us, as researchers, to conceptualize the factors that influence the development of vicarious trauma for FIFO providers. This framework provided guidance to understand the perceived risk and protective factors that influence the risk of developing vicarious trauma for FIFO mental health providers and the social and cultural context in which they work.

## Results

**THEME 1: Vicarious trauma is a risk associated with working in communities with high rates of trauma**

In the first theme, mental health providers identified exposure to trauma as an occupational hazard of their work as counsellors in Inuit Nunangat. Many participants described the risks associated with providing counselling services in small Inuit communities where members have experienced trauma. Rebecca stated,

Just the constant exposure to trauma, right? It’s an occupational hazard … it’s a small community. You have to manage being discreet and neutral in the community because you’re carrying so many people’s stories. You’re at high risk [of developing vicarious trauma] for sure.

Rebecca described the impacts of working in a community on a longer-term FIFO contract:

I feel quite exhausted at the end of the week because of the concentration of pain and the energy that it takes me to show up professionally and interact with a number of different professionals that I don’t have a history with.

In comparison, Susan shared her experience of working in a crisis response role and the challenges that this short-term position entailed. Susan recognized the potential of being triggered by the trauma that has cumulated from the lack of mental health care in some communities despite the overwhelming need for counselling services. Susan explained,

We know some of these deployments can be triggering … we had a couple of counsellors come in to respond to a suicide and while they were there, someone else committed suicide, and then there was an [type of accident] accident and [number of people] people died. The whole community was just completely overwhelmed with everything, and it’s really hard not to get overwhelmed yourself because you’re in the middle of that. People just offload on you, and it’s sometimes people who haven’t had the opportunity for this kind of counseling when there’s no crisis, so they also have a lot of pent up trauma or emotion or courage or other experiences that all get triggered and come out, so it’s huge what comes to our counsellors.

Whether service providers were in the community for short- or long-term contracts, all participants agreed that there is a risk for the development of vicarious trauma.

**THEME 2: Establishing individual and organizational strategies to reduce risk of vicarious trauma may improve FIFO providers’ well-being and career longevity**

While mental health service providers recognized the risk of developing vicarious trauma, they also identified supports and boundaries that they believe are required to promote personal well-being and career longevity. Carol explained her personal strategies when she is working in northern communities:

You have to be really self-aware and [have] excellent self-care. I always find a place that I can work-out in a community. And having the debriefing, open honest debriefing with one of the associates [within NCTS] to work through that, is really important. Because sometimes you can’t just say sorry, I can’t work with you. That’s not always an option.

Rachel also stated that debriefing with counsellors is essential:

I think it’s [debriefing with other counsellors] part of the job. It’s really, really important, because otherwise it [trauma] can be carried in their bodies and it’s just important to keep clear and keep healing and be present. We’re not immune. Counsellors are not immune to this.

Rebecca also emphasized the importance of self-care and identified physical activity as one of her strategies. Rebecca remarked, “I’d ride my bike to the hospital in the summer in [the community], you know so exercise was great.” Beyond physical activity, Rebecca also engaged in other self-care strategies such as massage therapy with a “massage therapist who did trauma massage therapy.”

James concurred with the other providers’ remarks regarding self-care and extended the discussion to include the significance of providers having their own therapist, often located in the South. He asserted,

Make sure that you are caring for yourself in healthy ways as opposed to unhealthy ways that includes alcohol and drugs. Exercise, and having your support network so friends and family but also your own counselor that you can call whenever you’re stressed to get specific strategies for the specific symptoms of PTSD that you may be facing, feeling or thinking.

Diane agreed that a strong support system is essential, and she emphasized the role that organizational support can play as a crucial aspect of self-care:

Having that support to process, debrief, release and knowing how to do that for yourself. At NCTS there’s strong support to be well within your work as a counselor and so that might mean that I need to talk to my own supervisor and just say I’m really struggling with the situation that I’m in front of right now and I feel like the organization supports that well, but also, I need to take that up as a counselor and be aware that this is a real thing that happens. Overtime you can, if you don’t take care and process continuously, fall into vicarious trauma, and compassion fatigue or burn out. I guess it’s the awareness that it definitely can happen. I guess as counselors we always need to have a supervisor … who we check in with about our role as the counselor and … when [you’re] beginning to feel signs of burnout or anything like that, it kind of keeps you in check.

In addition to self-care, personal and/or personnel support, participants discussed the importance of boundaries related to caseload management. Rachel described a strategy she employs in which she manages her caseload and sets boundaries for the type of cases she take on while engaging in other previously mentioned self-care approaches: “I sort of titrate my involvement with the traumatic situations and so I don’t load myself up too much. I basically make sure that I pace myself in terms of taking those types of cases.” Tanya found that limiting time within communities is a boundary that is essential. She remarked, “I don’t stay there too long, like, I usually like not to stay more than 10 days or 14 days, the most.” The importance of engaging in self-care strategies and setting boundaries was highlighted by all participants as crucial aspects of their practice that reduce the impact of vicarious trauma.

**THEME 3: FIFO models of care may offer protective benefits for mental health service providers against vicarious trauma**

Many providers credited the FIFO model of care as a potential protective factor against their own traumatization. David explained,

Honestly, I think that we are lucky in a sense, if we’re doing this kind of work, where we’re fly-in and fly-out. We have the opportunity to debrief with Northern Counselling support people and admin people. And then of course once we’re back in the South, we have the opportunity to access our own mental health services and our own support networks. And so, I think there’s lots of it [support] available once you’re out of those communities. When you’re in those communities, I don’t think there is much available at all. But again, we’re not there for a long period of time. So, in a way, it makes a huge difference when you know you’re leaving.

David described the benefits of having the ability to leave communities so that he can decompress, a strategy that he associated with mitigating the risk of vicarious trauma. Rachel echoed this statement and suggested that FIFO work offers health service providers who live in communities an opportunity to recover:

I think there’s certainly a danger of those things [vicarious trauma] happening, but I think in some ways it’s mitigated a little bit by the fly-in fly-out model. Because we’re in there, we deal with it for a while, and then we’re gone. And we get a period of time to recover.

Rachel predicted that the prevalence of trauma among other professionals in the community was higher due to the inability to have time to recover: “The school staff that I was dealing with, the occurrence of PTSD within that school [staff], I would probably tag it around 40%. And then other simple trauma and other types of just stress, I would say was close to 90% to a 100% of the staff were experiencing that.”

In line with the aforementioned comments, Tanya described the benefits of being able to go home and for minimizing the amount of time spent in the community and the exposure to traumatic experiences. She stated:

Usually, we stay for a week or two. Sometimes, some people stay for three weeks. Myself, I don’t want to stay too long, because it’s also hard on ourselves, right, and we have lives here [in the South], as well. So – and I find for our home and for health, it’s good not to stay too long. So, we can still be objective, and we can be refreshed after a while.

Rachel felt that the FIFO model of care may act as a protective factor against the development of vicarious trauma, and she expanded the discussion to include what puts FIFO providers at risk. Rebecca agreed, arguing that the FIFO model enables providers to benefit from breaks and keep returning, thus continuing to build relationships within the community:

That’s a benefit of fly-in fly-out. For a couple of months at a time, the relationship building is there. But the person who’s there, you know living in the same building in which they offer services [e.g., health centre], they can make sure to get some distance [by flying out] - you know, and not let their whole consciousness be around just their work.

According to providers, the FIFO model itself may serve as a strategy to manage the risk of vicarious trauma and can alleviate stress not only for the travelling provider.

## Discussion

Through this research, we aimed to provide insight into the factors that influence the experience of vicarious trauma for FIFO mental health providers in Inuit communities and to propose how FIFO models of care may affect the risk of vicarious traumatization. These conceptualizations were informed by the CSDT framework, which we employed to examine how providers perceive risk and protective factors of developing vicarious trauma, and how they adapt to trauma.This study advances the current literature on vicarious trauma among mental health providers in northern Canada (O’Neill, 2010; O’Neill et al., 2016) with a focus on the Inuit Nunangat and FIFO contexts. Overall, we found that participants acknowledged the risks of developing vicarious trauma as a result of working with highly traumatized clients in Inuit communities. As a result, the providers in our study highlighted the importance of developing self-care routines, establishing boundaries, and maintaining supports to promote well-being and career longevity. The providers acknowledged the FIFO model of care as a potential protective factor from vicarious traumatization. These research findings offer opportunities to inform future practice and policy for the delivery of mental health services to Inuit Nunangat.

## Perceived risk

In alignment with the CSDT framework, the mental health service providers in our study agreed that their engagement with trauma work makes them more vulnerable to the negative effects of vicarious trauma. Providers in our study acknowledged that their self-capacitiescan be affected by engaging with traumatized clients, which is characteristic of CSDT as described by McCann and Pearlman (1990). Similar to other research on vicarious trauma (Quitangon, 2019), providers in our study recognized the inherent risk to their mental health when working with traumatized clients and communities. Although experiences of vicarious trauma can happen in any setting where trauma therapy is being delivered, Inuit have experienced, and continue to experience, severely traumatic events that may affect those who empathetically engage with this population. The providers in this study described the potential risks of developing vicarious trauma based on the type of contract (short-termor long-term) and thus the duration of time they spent in the communities. A typical workday may vary for providers depending on their role, and it can include long workdays for crisis response workers who attempt to see as many clients as possible while in community, compared to providers who work during regular business hours in long-term positions. The providers who assumed a short-term, crisis response role acknowledged the potential of risk of traumatization in the community due to the overwhelming need for mental health services for a large number of clients over a short period of time. These findings align with the work of scholars who have noted that heavier caseloads and constant exposure to trauma stories put providers at a heightened risk for developing vicarious trauma (Bober & Regehr, 2006; Pearlman & Mac Ian, 1995). Providers in our study affirmed that the prolonged exposure to trauma in a small community setting for an extended period of time can be overwhelming for them. In an article addressing health care provider turnover in Nunavut, Cherba et al. (2019) highlighted the need for Inuit to retain short-term locum physicians. They argued that the high turnover of health care personnel impacts the quality of care for residents in Nunavut through low patient satisfaction, poorer health outcomes, and negative effects on community-provider relationships. Cherba et al. (2019) noted the specific impact that high turnover has on mental health services, citing the lack of continuity in provision of services as a potential cause of critical symptoms being missed. Although discussed in a slightly different context (i.e., primary care provision), the need to ensure the mental well-beingof FIFO providers is crucial to optimizing care for community members.

## Prevention strategies

Despite being in Inuit communities for relatively short contracts, the participants in our study noted that establishing strategies to prevent negative emotional responses to trauma work is of utmost importance. Prevention approaches are well reported in the literature, which suggests that engaging in different strategies can affect manifestations of vicarious trauma (Cohen & Collens, 2013; Finklestein et al., 2015; Pearlman & Mac Ian, 1995). Engaging in self-care behaviours has been described as a way for individuals to regulate emotions and experiences, and it is required to provide high-quality services (Cohen & Collens, 2013).

All providers in this study emphasized self-care as crucial to their well-being as FIFO workers. Some of the self-care strategies the providers used included physical activity, massage therapy, and speaking with family, friends. Providers in our study also noted that access to external supports on a professional and organizational level is key, including debriefing with fellow associates and supervisors. Michalopoulos and Aparicio (2012) described increased social support as a professional variable that could predict less severe vicarious trauma. Furthermore, O’Neill et al. (2016) reported that consistent clinical supervision could buffer the effects of trauma. In addition to these supports, providers in this study also described setting boundaries as an essential aspect of their practice such as reducing caseload and time spent in community. On an organizational level, Pearlman and Mac Ian (1995) proposed that balancing personal and work life can mitigate negative impacts of trauma, while Bober and Regehr (2006) found that distributing caseload among therapists to limit exposure to trauma can significantly reduce the impact of trauma. Participants in our study reported using both of these strategies while working FIFO contracts, and they identified them important elements for organizations to consider when distributing workload to employees. Engagement with prevention strategies is important for FIFO mental health service providers to avoid symptomatic adaptions or disruptions to previous belief systems that occur most commonly within the five components of self described by CSDT (Trippany et al., 2004) as a result of vicarious traumatization.

## FIFO model of care as a mitigator vicarious trauma

To mitigate the risk of vicarious trauma noted by providers, participants noted that in addition to the strategies they engage in, the FIFO model of care serves as an approach to reduce the risk of traumatization. Through FIFO contracts, providers are able to limit the amount of time they spend in communities and, in turn, their exposure to trauma stories. Through the CSDT framework, the FIFO model of care could be employed to manage the risk of emotional, physical, and spiritual transformations through reduced exposure to traumatic material (Clemans, 2005). Although their exposure to trauma may be heightened while in a community, being employed on a FIFO basis allows providers to exit the community after a short amount of time. This gives providers the ability to engage in self-care routines such as exercise and time spent with friends, which, according to Harrison and Westwood (2009), contribute towards well-being. In a study looking at the experiences of FIFO and drive-in and drive-out services with remote psychologists in Australia, researchers found that working on a FIFO basis in a rural community was associated with avoidance of burnout (Sutherland et al., 2017).Although burnout is a different construct than vicarious trauma (O’Neill, 2010), the psychologists in Sutherland et al.’s (2017) study pointed to the advantage of being able to seek support outside of the affected rural community when a tragedy occurs (Sutherland et al., 2017).

The findings of this PAR process were used to identify areas for action and change. As such, NCTS is in the process of revising policies and practices to further prioritize the mental health and well-being of its employees who are mental health service provides to clients in Inuit Nunangat, which will also serve to enhance continuity of care and thus better care for Inuit. Specifically, it is doing the following: 1) Exploring the optimal amount time spent in communities that would allow for counsellors to develop relationships with community members and also maintain their mental health; 2) strengthen policies, procedures, and internal structures to encourage discreet and accessible pre- mid- and post-deployment debriefing with fellow associates or supervisors and other forms of self-care strategies.

## Limitations

As with all research, our study has limitations. The aim of this paper was to focus on the experience of FIFO service providers; however, we acknowledge that the most important perspectives are those of Inuit community members. Regardless of what is best for mental health service practitioners who work in Inuit Nunangat, the needs of Inuit are of utmost importance. The FIFO model presented in this study has clear benefits for providers, but its benefits for community members may be limited. While ensuring that FIFO mental health practitioners are mentally well enough to continue FIFO work is of benefit to Inuit in terms of providing some continuity of care, permanent, resident, Inuit practitioners would likely provide the greatest benefit to Inuit (Cherba et al., 2019; Inuit Tapirit Kanatami, 2014). Further, upstream, Inuit-led solutions to address past and current traumatic colonial practices that continue to put Inuit at heightened risk for poor mental health are urgently needed. Future research should also consider the the ways in which different cultural identities between southern-based service providers (who are typically non-Inuit) and Inuit clients may impact mental health service delivery.

The scope of this study was limited as participants were all employed by one organization, Northern Counselling and Therapeutic Services. Though their experiences may be directly related to other FIFO providers, the professional and organizational support for service providers will vary across organizations. This is an important consideration as these variables play an important role in the development of vicarious trauma (Cohen & Collens, 2013; Finklestein et al., 2015; Michalopoulos & Aparicio, 2012; Trippany et al., 2004). Although the sample size was small and the participants were employed within one organization, this study provides useful insights into the FIFO mental health service provision and the risk of developing vicarious trauma. In the future, researchers might also consider how FIFO workers spend their time while outside of work in the Inuit communities, and if they engage in other activities that may contribute to the development or mitigation of vicarious trauma.

## Conclusion

Our research adds a nuanced perspective to the vicarious trauma literature to include the perspective of FIFO mental health service providers who deliver services to communities in Inuit Nunangat. The findings from this study confirm and extend prior research to suggest that FIFO models of care may reduce the impact of vicarious trauma by allowing providers to reduce their caseload and limit their exposure to traumatic work. When discussing the perceived risk of vicarious trauma for FIFO mental health providers, it is important to consider the synergistic effects of three elements: 1) working in a remote community with limited supports, 2) working between cultures with different worldviews, and 3) heightened degrees of trauma experienced by Inuit. These three elements increase the vulnerability of developing vicarious trauma for FIFO providers and should be considered when implementing policies and practices. Furthermore, this research provides valuable theoretical contributions to the CSDT framework by highlighting the risks of developing vicarious trauma for FIFO mental health providers. By applying the CSDT model to their own experiences, professionals can prevent negative consequences of vicarious trauma and encourage self-care (Trippany et al., 2004). Although there is a significant amount of literature that has investigated ways to predict the development of vicarious trauma, this study acknowledges the potential benefits of the FIFO model for mental health service providers.
